# Spraying Hatching Eggs with Clove Essential Oil Does Not Compromise the Quality of Embryos and One-Day-Old Chicks or Broiler Performance

**DOI:** 10.3390/ani11072045

**Published:** 2021-07-08

**Authors:** Gabriel da Silva Oliveira, Sheila Tavares Nascimento, Vinícius Machado dos Santos, Bruno Stéfano Lima Dallago

**Affiliations:** 1 Faculty of Agronomy and Veterinary Medicine (FAV), University of Brasília, Brasília 70.910-900, Brazil; gabriels.unb@gmail.com (G.d.S.O.); sheilatn@unb.br (S.T.N.); dallago@unb.br (B.S.L.D.); 2 Laboratory of Poultry Science, Campus Planaltina—Federal Institute of Brasília, Brasília 73.380-900, Brazil

**Keywords:** broilers, chick quality, clove essential oil, embryo development, hatching eggs

## Abstract

**Simple Summary:**

Studies on natural sanitizers for potentially safe hatching of eggs are essential. In this context, the objective of this study was to evaluate whether sanitizing hatching eggs with clove essential oil in the preincubation phase affects broiler performance (body weight, body weight gain, feed consumption, feed conversion ratio, and survivability). Furthermore, the effects of the oil on the hatch window and quality of embryos and one-day-old chicks were investigated. In this study, clove essential oil did not compromise the quality of the birds or the post-hatch performance.

**Abstract:**

The objective of this study was to evaluate whether sanitizing hatching eggs with clove essential oil in the preincubation phase affects broiler performance and influences the hatch window and quality of embryos and one-day-old chicks. Hatching eggs (*n* = 1280; mean weight = 58.64 ± 0.49 g) from a batch of 37-week-old broiler breeder hens of the CPK (Pesadão Vermelho) lineage were randomly distributed into four treatments in the preincubation phase. The treatments consisted of three different sanitization procedures (spraying with grain alcohol, spraying with clove essential oil, and fumigation with paraformaldehyde) and a control treatment (nonsanitized). The lengths of the embryos and one-day-old chicks (one of the parameters used to assess bird quality) were not significantly different among the treatments, with means of 15.30 ± 1.41 and 18.37 ± 0.76 mm, respectively. Body weight, body weight gain, feed consumption, and feed conversion rate in different rearing periods did not differ significantly among the treatments. However, there was a significant difference in the percentage of survivability during the initial period (1 to 28 days) among the treatments. In conclusion, clove essential oil treatment did not negatively affect the quality of embryos and one-day-old chicks or the performance of broilers.

## 1. Introduction

The surface of the shells of newly laid eggs can be colonized by distinct microorganisms, which can negatively affect the productive and economic capacity of poultry farming by increasing embryo mortality rates and reducing the quality of one-day-old chicks [1,2,3,4]. Therefore, to mitigate mortality and reduce the eggshell microbiota that are potentially pathogenic to embryos, the poultry industry adopts a variety of strategies, including the implementation of biosafety practices.

The sanitization of hatching eggs is a common biosafety practice performed in farms and hatcheries and mainly involves the use of paraformaldehyde [5,6,7], a product that, although effective against microorganisms, is carcinogenic and teratogenic [8,9,10], presenting a risk to the health of chicken embryos and chicken egg handlers [8,10,11]. Therefore, researchers have sought to develop and evaluate potentially safe products that reduce the pathogenic microbial load of hatching eggshells, with the goal of minimizing impacts on human and animal health.

The application of clove essential oil in sanitizing formulations used on hatching eggs is safe and recommended [3]. Studies have shown that clove essential oil is effective in reducing the microbial load of hatching eggshells, provides good results in terms of incubation performance, and improves the quality of neonate chicks [3,12]. This oil is an aromatic hydrophobic extract of *Syzygium aromaticum* (family: Myrtaceae) and is mainly composed of phenylpropanoids, such as eugenol (C_10_H_12_O_2_), which constitutes 90% of the oil and is the main compound responsible for its antimicrobial activity [13,14].

Considering that clove essential oil is a promising sanitizing compound for hatching eggs and that no studies have investigated the performance of broilers hatched from eggs treated with this oil, the objective of this study was to evaluate whether the sanitization of hatching eggs with clove essential oil in the preincubation phase affects broiler performances. Furthermore, this study investigated whether this oil affects the hatch window and the quality of embryos and one-day-old chicks.

## 2. Materials and Methods

### 2.1. Experimental Procedure

Hatching eggs (*n* = 1280; mean weight = 58.64 ± 0.49 g) from 37-week-old broiler breeder hens of the CPK line (known as Pesadão Vermelho) were randomly distributed into four treatments in the preincubation phase. CPK is a slow-growing broiler breeder line with red plumage that is active, resistant, and adapted to free-range systems. The treatments consisted of three different sanitization procedures (spraying with grain alcohol, spraying with clove essential oil, and fumigation with paraformaldehyde) and a control treatment (nonsanitized). The experimental protocol was approved by the Animal Use Ethics Committee of the University of Brasília (Document number 33/2019).

### 2.2. Sanitizers and Sanitization Methods

#### 2.2.1. Nonsanitized

The eggs used for this treatment were not subjected to any sanitization process.

#### 2.2.2. Grain Alcohol

Grain alcohol (93.5%) (Cromoline Química Fina, Diadema, São Paulo, Brazil) served as the carrier vehicle for clove essential oil; therefore, it was tested to ensure that there was no synergism. Eggs were sprayed individually with grain alcohol using a manual sprayer. After spraying, the eggs were placed in sterile trays (30 to 50 min) to dry at room temperature. The trays were sterilized by ultraviolet light at 254 nm for 15 min in a laminar flow cabinet (OptiMair, ESCO, Horsham, PA, USA) at a microbiology laboratory (Federal Institute of Brasília, Planaltina, Federal District, Brazil).

#### 2.2.3. Clove Essential Oil

The essential oil was extracted from dried clove flower buds by hydrodistillation according to a method adapted from Ascenção and Filho [15] using the Clevenger extractor system (Vidrolabo, Poá, São Paulo, Brazil). Subsequently, the oil was diluted in 93.5% grain alcohol at a concentration of 0.39% (*p*/*v*) [3]. The spraying and drying procedures used were similar to those performed on eggs treated with grain alcohol.

#### 2.2.4. Paraformaldehyde

In this treatment, eggs were sanitized by fumigation for 20 min with 6 g/m^3^ paraformaldehyde volatilized on a metal plate in a sanitization chamber (temperature: 25 °C; relative humidity: 70%), according to the guidelines of the commercial hatchery.

All sanitization processes were conducted in a commercial hatchery (Planaltina, Federal District, Brazil) 20 min after egg collection.

### 2.3. Storage and Incubation

The eggs were stored for a period of three days in a poultry science laboratory (Federal Institute of Brasília, Planaltina, Federal District, Brazil). During storage, the temperature was maintained between 16 and 18 °C, and the relative air humidity was maintained between 55 and 60%. After storage, the egg trays were weighed and distributed in different trays in four single-stage setters (Luna 480, Chocmaster, Curitiba, Paraná, Brazil). For each treatment, four incubation trays were used, with one tray in each setter (tray position, and therefore, the treatment, was distributed randomly) for a total of 320 eggs for each treatment. The setters were sanitized with a lysoform-based (SC Johnson, Racine, WI, USA) liquid sanitizer before incubation according to the manufacturer’s guidelines. The temperature of the incubation room was maintained between 22 and 24 °C, and the relative humidity was maintained between 50 and 55%. All microclimatic variables were monitored by thermohygrometers (608-H1, Testo, Campinas, São Paulo, Brazil).

From the beginning of incubation until the 18th day, the mean temperature and relative humidity of the setters were 37.7 °C and 60%, respectively. During this same period, the eggs were turned by a 45° angle every hour. On the eighth day, all eggs were subjected to candling and the infertile eggs were removed and opened to confirm infertility according to Aviagen [16]. No egg was replaced. On the 19th day, the incubation trays were weighed again, and the setters were operated at a mean temperature and relative humidity of 36.6 °C and 65%, respectively. The incubation process was terminated on the 21st day.

### 2.4. Hatch Window

The egg hatching time was defined by video recordings using four infrared bullet cameras (VHD 1010B G4, Intelbras, Campinas, São Paulo, Brazil). After 462 h of incubation, the number of chicks that hatched was recorded every 6 h. The chicks were counted, weighed, and removed from the setters so that the cameras maintained good visibility. The hatch window comprised the period between the first and last hatched chick in each tray.

### 2.5. Length and Weight of Embryos and One-Day-Old Chicks and Weight of Residual Yolk, and Relative Organ Weight

On the 18th day of incubation, 15 eggs from each treatment were randomly selected and removed from the setters. The eggs were opened, embryo length was recorded and, then, the embryos were euthanized by cervical dislocation. The length (mm) of the embryo, wing, beak, and leg were measured with a 0.001-mm precision digital calliper (Mitutoyo, Suzano, São Paulo, Brazil). The embryo, residual yolk, heart, liver and gallbladder, proventriculus and gizzard, breast, and intestine were weighed (g) using a 0.0001-g precision analytical scale (Gehaka, São Paulo, São Paulo, Brazil). Relative organ weights were calculated as a percentage of the weight of the embryo without residual yolk. On the first day post-hatching, 15 one-day-old chicks from each treatment were randomly selected and the same procedures used for embryo measurements were conducted. However, the relative organ weights were calculated as a percentage of the chick weight. These analyses were used as parameters to assess the quality of birds.

### 2.6. Broiler House and Management

After 21 days of incubation, 90 healthy chicks (45 females/45 males; sexed according to the wing feather characteristics) from each treatment with similar weights (mean = 40.23 ± 0.72 g) were subdivided into five replicates. Each replicate group (18 birds = 9 females/9 males) was randomly housed in a 2.40 m² pen (experimental unit) equipped with feeders and drinkers suitable for each growout period, in addition to poultry bedding made of rice straw. The birds had ad libitum access to feed and water and received the same diet, which was formulated as follows according to Prado [17] for each rearing period: initial, corn 63%, soybean meal 33%, and minerals and vitamins 4%; growth, corn 68%, soybean meal 28%, and minerals and vitamins 4%; final, corn 82%, soybean meal 14%, and minerals and vitamins 4%. The feed was supplied twice a day. The vaccination schedule followed the guidelines in the free-range chicken farming manual [18].

During the first 14 days, infrared lamps provided heat to the birds, which were exposed to 24 h of light. From the 15th day, a lighting schedule of 16 h of light:8 h of dark was initiated. The birds were subjected to similar temperature and humidity conditions throughout the study. The broiler house was equipped with a ventilation system with two manually operated fans, nebulization, and side curtains.

The birds (pen weight) and wasted feed from each experimental unit were weighed weekly on a precision scale (Triunfo, São Paulo, São Paulo, Brazil) until the 70th day; however, only the weights of days 28, 56, and 70 were used to evaluate performance (body weight, weight gain, feed consumption, and feed conversion), representing the three rearing periods (initial (day 1 to day 28), growth (day 29 to day 56), and final (day 57 to day 70)]. The scale was observed by 15 s approximately. During this period, the highest and lowest values were recorded and then the mean between these values was used as weight of birds. Mortality was recorded throughout the experimental period to calculate survivability. Birds were not replaced in the house; moreover, all dead birds were weighed so that it was possible to adjust the feed consumption and feed conversion during the period.

### 2.7. Experimental Design and Statistical Analysis

The experiment followed a completely randomized design with four treatments (nonsanitized, grain alcohol, clove essential oil, and paraformaldehyde). In the analysis of embryo and chick quality, each embryo and chick was considered a replicate. The analysis of posthatching performance was based on five replicates per treatment, in which each pen of 18 birds constituted a replicate. All analyses were performed with SAS Studio University Edition (Inst. Inc., Cary, NC, USA). The data were analyzed by analysis of variance (PROC GLM), and means were compared using Tukey’s test. The hatching curves were subjected to survival analysis using the PROC LIFETEST command and the Kaplan–Meier method combined with log-rank analysis with subsequent comparison by Tukey’s test. The hatch window (period between the first and last hatching) was compared by the Kruskal–Wallis test using the PROC NPAR1WAY procedure. Statistical significance was considered at *p* < 0.05 for all tests.

## 3. Results

### 3.1. Hatch Window

**Figure 1:**
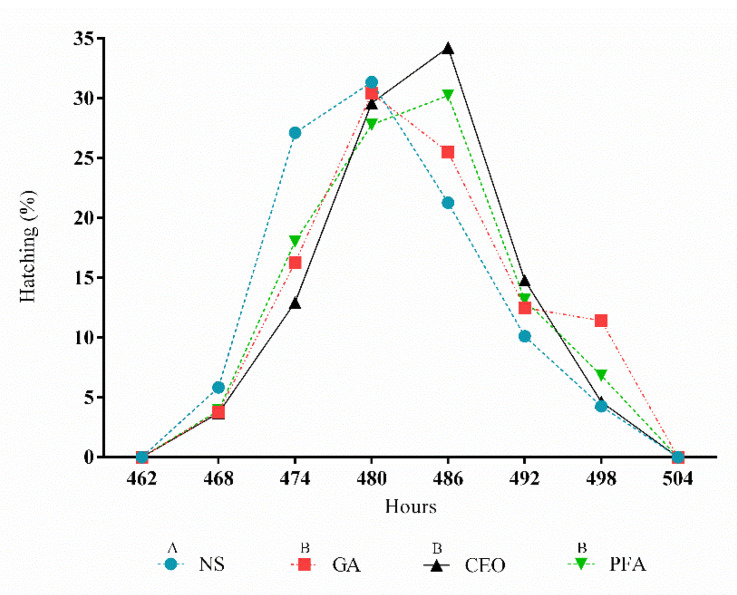
Hatching curves for different sanitization treatments. NS, nonsanitized; GA, grain alcohol; CEO, clove essential oil; PFA, paraformaldehyde. ^A,B^ Curves with different letters differ significantly (*p* < 0.05).

Chick hatching began between hours 462 and 468 and ended between hours 492 and 498 for all treatments (Figure 1). There were no significant differences in the hatch window among the treatments (*p* = 0.8348). However, the hatching curves of the grain alcohol, clove essential oil, and paraformaldehyde treatments differed (*p* = 0.006) from those of the nonsanitized treatment (Figure 1).

### 3.2. Quality of Embryos and One-Day-Old Chicks

The lengths of embryos (mean = 15.30 ± 1.41 mm), wings, beaks, and leg at 18 days of incubation as well as the lengths of chicks (18.37 ± 0.76 mm), wings, beaks, and legs at 1 day of age were not significantly different (*p* > 0.05) among the treatments (Table 1 and Table 2).

The absolute weights of the embryo (mean = 29.75 ± 3.02 g) and residual yolk at 18 days of incubation (11.79 ± 2.58 g) as well as the weight of chicks (40.44 ± 4.20 g) and residual yolk at 1 day of age (3.44 ± 1.05 g) were not affected (*p* > 0.05) by the sanitization treatments (Table 1 and Table 2).

The relative organ weights of the embryos at 18 days of development and those of one-day-old chicks were similar (*p* > 0.05) among the treatments, except for the relative weights of the liver and gallbladder, breast, and intestine of the embryos at 18 days of age (*p* < 0.05) (Table 1 and Table 2). The weights of the liver and gallbladder, breast, and intestine of the embryos subjected to the paraformaldehyde treatment were significantly higher than those of the embryos subjected to the grain alcohol treatment; however, the values for the three treatments did not differ from those for the nonsanitized treatment.

### 3.3. Broiler Performance

Body weight, body weight gain, feed consumption, and feed conversion ratio in the different rearing periods did not differ significantly (*p* > 0.05; Table 3) among the treatments; however, there was a significant difference (*p* = 0.0397) in the percentage of survivability during the initial period (1 to 28 days). The survivability of birds hatched from eggs treated with clove essential oil (97.96%) did not differ significantly from that of birds hatched from eggs treated with paraformaldehyde (96.94%), but it was significantly higher than that of birds in the grain alcohol (92.84%), and nonsanitized (92.48%) treatments.

## 4. Discussion

Essential oils have been used as effective sanitizers for hatching eggs [3,19,20]. Positive effects on the sanitization of hatching eggs with essential oils, such as clove essential oil, have been described in terms of both antimicrobial efficiency and incubation performance and in terms of bird quality [3,12]. In this sense, to contribute to the results already described in the literature associated with the applicability of this oil for hatching eggs, the present study investigated the effects of clove essential oil on post-hatch performance, also considering the effect on the hatch window and bird quality.

### 4.1. Hatch Window

The mean hatch window was 27.75 h, corroborating the results described by Decuypere et al. [21], who reported a hatch window range of 24 to 48 h in commercial hatcheries. However, the mean hatching time of the nonsanitized eggs was 2.5 h shorter than the mean hatching time of eggs treated with grain alcohol, clove essential oil, or paraformaldehyde. In addition, Figure 1 shows that a high number of eggs in this treatment hatched earlier, before the middle of the hatch window period, which is in fact a disadvantage because it increases the duration that chicks remain in the hatcher, which consequently increases the likelihood of animal dehydration [22]. On the other hand, more eggs sanitized with clove essential oil and paraformaldehyde hatched in the middle of the hatch window period, indicating the importance of good sanitization of hatching eggs to maintain adequate embryonic development.

### 4.2. Quality of Embryos and One-Day-Old Chicks

The quality of embryos and one-day-old chicks is of great importance for poultry production. The lengths of embryos and one-day-old chicks were measured to evaluate the effects of the treatments on bird quality. Length measurements (Table 1 and Table 2) showed that the quality of the embryos and one-day-old chicks was not affected, confirming that the tested sanitizers did not negatively impact bird development, possibly because they did not alter the properties of the cuticle, since the application of sanitizers in the eggshell can affect the cuticle and alter the permeability of the eggshell and embryonic development [23].

The weights of the embryos and one-day-old chicks were also used to assess bird quality. The different sanitization treatments did not significantly alter the weights of the embryos and one-day-old chicks or the weights of the respective residual yolks (Table 1 and Table 2). The yolk is the main source of energy for the growth and maintenance of the body during embryonic development [24,25]. Therefore, the results suggest that the amount of nutrients absorbed from the yolk and converted into body tissue by the birds during development was proportional among the treatments, which may have contributed to the similar weights of these birds. In addition, the amount of residual yolk and the weight of embryos and one-day-old chicks are mainly affected by the age and lineage of the broiler breeder hens and the duration and conditions of storage and incubation [26,27,28]. This finding may explain the similarity among treatments for these variables in this experiment because all eggs were from broiler breeders of the same age and lineage, subjected to the same storage conditions and time, and exposed to the same incubation conditions.

The relative internal organ weights (Table 1 and Table 2) served as an indicator of the responses of the embryos and one-day-old chicks to the possible toxicity of the sanitizers. In this experiment, no macroscopic alterations, such as atrophy or hypertrophy, were observed in the internal organs. Therefore, it can be stated that the tested sanitizers did not negatively affect the development of organs during embryogenesis and the post-hatching period. The results suggest that clove essential oil at 0.39% is safe for embryos and does not negatively affect their survival, growth, or health. In addition, the results suggest that adequate sanitization of eggs with paraformaldehyde can avoid the adverse effects of this compound on the development of birds. The adverse effects of paraformaldehyde in chick embryos described in the literature, include malformations, low weight, and underdevelopment [8].

### 4.3. Broiler Performance

Fasenko et al. [29] did not observe significant differences in body weight or feed conversion between broilers from eggs sanitized with electrolyzed oxidizing water and those not sanitized during 39 days of growth, which is in accordance with our results. In this study, the percentage of survivability for the first 28 days of broiler rearing was higher in the clove essential oil treatment group than in the nonsanitized treatment group (Table 3). Although chick quality did not differ among the treatments in terms of weight and length, we can hypothesize that chicks from eggs sanitized with clove essential oil had a lower microbial load than chicks from eggs that were not sanitized, which may have resulted in greater survivability in the initial period in the clove oil treatment group. Therefore, studies that measure the internal microbial load of chicks from eggs sanitized with clove essential oil need to be carried out to support this hypothesis.

## 5. Conclusions

Clove essential oil treatment did not impair the hatch window, quality, or development of embryos and one-day-old chicks, or the performance parameters evaluated (body weight, body weight gain, feed consumption, feed conversion ratio, and survivability). Therefore, considering the factors evaluated here, this oil can be used as a sanitizer for incubating eggs safely for the birds.

## Figures and Tables

**Table 1:** Mean values for lengths of the embryo, wings, beaks, and legs, weight of the embryo without residual yolk and of the residual yolk, and the relative organ weight in the different sanitization treatments.

Items	Treatments					
	Nonsanitized	Grain Alcohol	Clove Essential Oil	Paraformaldehyde		
	Length (mm)				*p*	CV (%)
Embryo	15.44 ± 1.78	14.87 ± 1.20	15.75 ± 1.07	15.14 ± 1.59	*	9.21
Wing	2.65 ± 0.37	2.73 ± 0.35	2.84 ± 0.28	2.62 ± 0.33	*	12.38
Beak	1.11 ± 0.18	1.15 ± 0.15	1.18 ± 0.24	1.10 ± 0.14	*	14.91
Leg	3.96 ± 0.39	3.80 ± 0.46	4.14 ± 0.36	3.75 ± 0.70	*	13.10
	Weight (g)					
Embryo without residual yolk	29.85 ± 4.05	29.05 ± 1.94	29.57 ± 2.80	30.51 ± 3.27	*	10.20
Residual yolk	11.87 ± 2.23	11.48 ± 2.74	11.97 ± 1.82	11.82 ± 3.51	*	22.72
	Relative organ weight (%)					
Heart	0.67 ± 0.05	0.69 ± 0.08	0.74 ± 0.16	0.85 ± 0.14	*	55.11
Liver and gallbladder	1.84 ± 0.17 ^ab^	1.65 ± 0.14 ^b^	1.99 ± 0.12 ^ab^	2.16 ± 0.18 ^a^	0.0021	24.85
Proventriculus and gizzard	5.80 ± 0.43	5.05 ± 0.38	5.55 ± 0.41	5.64 ± 0.37	*	24.33
Breast	4.25 ± 0.22 ^ab^	4.06 ± 0.22 ^b^	4.33 ± 0.18 ^ab^	4.82 ± 0.21 ^a^	0.0039	15.42
Intestine	2.71 ± 0.28 ^ab^	2.44 ± 0.30 ^b^	2.80 ± 0.28 ^ab^	3.05 ± 0.62 ^a^	0.0134	44.41

^a,b^ Means in the same row with different superscript letters differ significantly (*p* < 0.05). * nonsignificant; CV, coefficient of variation.

**Table 2:** Mean values for lengths of the chick, wings, beaks, and legs, weight of the chick and residual yolk, and relative organ weight in the different sanitization treatments.

Items	Treatments					
	Nonsanitized	Grain Alcohol	Clove Essential Oil	Paraformaldehyde		
	Length (mm)				*p*	CV (%)
Chick	18.53 ± 0.59	18.13 ± 0.96	18.63 ± 0.72	18.17 ± 0.75	*	4.40
Wing	3.37 ± 0.19	3.29 ± 0.37	3.55 ± 0.52	3.34 ± 0.31	*	10.72
Beak	1.03 ± 0.08	1.07 ± 0.09	1.09 ± 0.09	1.06 ± 0.12	*	9.10
Leg	4.18 ± 0.13	4.13 ± 0.26	4.28 ± 0.20	4.12 ± 0.25	*	5.17
	Weight (g)					
Chick	40.61 ± 3.23	40.04 ± 5.28	40.98 ± 4.83	40.14 ± 3.45	*	10.58
Residual yolk	3.38 ± 0.78	3.46 ± 1.18	3.43 ± 1.20	3.47 ± 1.02	*	30.77
	Relative organ weight (%)					
Heart	0.81 ± 0.09	0.80 ± 0.09	0.88 ± 0.11	0.90 ± 0.10	*	28.51
Liver and gallbladder	3.42 ± 0.28	3.25 ± 0.32	3.32 ± 0.37	3.29 ± 0.31	*	23.91
Proventriculus and gizzard	6.57 ± 0.52	6.39 ± 0.44	6.98 ± 0.61	6.72 ± 0.56	*	19.95
Breast	2.19 ± 0.27	2.17 ± 0.25	2.27 ± 0.25	2.19 ± 0.26	*	29.76
Intestine	7.36 ± 0.81	7.04 ± 0.58	7.76 ± 1.10	7.20 ± 0.82	*	28.37

No significant differences existed between means (*p* > 0.05). * nonsignificant; CV, coefficient of variation.

**Table 3:** Mean values for the body weight, body weight gain, feed consumption, feed conversion ratio, and survivability of broilers from eggs sanitized with different sanitizers.

Parameters	Treatments					
	Nonsanitized	Grain Alcohol	Clove Essential Oil	Paraformaldehyde	*p*	CV (%)
	1 to 28 days (initial period)					
Body weight (g)	541.68 ^A^	544.45 ^A^	550.51 ^A^	553.31 ^A^	*	3.71
Body weight gain (g)	501.07 ^A^	513.50 ^A^	510.06 ^A^	514.44 ^A^	*	4.27
Feed consumption (g)	861.72 ^A^	875.59 ^A^	861.34 ^A^	871.58 ^A^	*	4.86
Feed conversion ratio	1.720 ^A^	1.704 ^A^	1.689 ^A^	1.689 ^A^	*	3.21
Survivability (%)	92.48 ^Bb^	92.84 ^Bb^	97.96 ^Aa^	96.94 ^Aab^	0.0397	4.01
	29 to 56 days (growth period)					
Body weight (g)	1975.88 ^B^	2015.84 ^B^	2002.92 ^B^	2010.38 ^B^	*	2.11
Body weight gain (g)	1434.20 ^B^	1461.38 ^B^	1452.41 ^B^	1457.07 ^B^	*	2.18
Feed consumption (g)	3821.57 ^B^	3907.88 ^B^	3896.01 ^B^	3795.28 ^B^	*	6.67
Feed conversion ratio	2.664 ^B^	2.674 ^B^	2.683 ^B^	2.603 ^B^	*	6.36
Survivability (%)	100.00 ^A^	100.00 ^A^	100.00 ^A^	100.00 ^A^	*	0.00
	57 to 70 days (final period)					
Body weight (g)	2667.92 ^C^	2742.08 ^C^	2731.84 ^C^	2728.97 ^C^	*	2.36
Body weight gain (g)	692.04 ^C^	726.26 ^C^	728.75 ^C^	718.59 ^C^	*	5.38
Feed consumption (g)	2643.98 ^C^	2842.03 ^C^	2741.80 ^C^	2745.06 ^C^	*	6.49
Feed conversion ratio	3.830 ^C^	3.915 ^C^	3.765 ^C^	3.819 ^C^	*	5.71
Survivability (%)	98.82 ^A^	98.82 ^A^	98.82 ^A^	100.00 ^A^	*	1.82
*P*	<0.0001	<0.0001	<0.0001	<0.0001		
1 to 70 days (overall period)						
Total survivability (%)	97.10	97.22	98.93	98.98	*	3.33

^a,b^ Means in the same row with different lowercase letters differ significantly (*p* < 0.05). ^A,B,C^ Means in the same column with different uppercase letters differ significantly (*p* < 0.05). * nonsignificant; CV, coefficient of variation.

## Data Availability

Data sharing not applicable.
